# Identification of risk factors associated with national transmission and late presentation of HIV-1, Denmark, 2009 to 2017

**DOI:** 10.2807/1560-7917.ES.2021.26.47.2002008

**Published:** 2021-11-25

**Authors:** Maarten van Wijhe, Thea K Fischer, Jannik Fonager

**Affiliations:** 1 Department of Science and Environment, Roskilde University, Roskilde, Denmark; 2 Virus Research & Development Laboratory, Virus & Microbiological Special Diagnostics, Statens Serum Institut, Copenhagen, Denmark; 3 Department of Research, University hospital of Nordsjælland, Hillerød, Denmark; 4 Department of Public Health, University of Copenhagen, Copenhagen, Denmark

**Keywords:** HIV-1, surveillance, cluster analysis, late presenters, phylogenetic analysis, pre-exposure prophylaxis

## Abstract

**Background:**

Despite availability of pre-exposure prophylaxis (PrEP), the incidence of HIV-1 in Europe remained stable the past decade. Reduction of new HIV-1 infections requires more knowledge about the profiles of high-risk transmitters and late presenters (LP).

**Aim:**

We aimed to investigate risk factors associated with HIV-1 transmission clusters and late presentation with HIV-1 in Denmark.

**Methods:**

Blood samples and epidemiological information were collected from newly diagnosed HIV-1 patients between 2009 and 2017. We genotyped pol genes and performed phylogenetic analyses to identify clusters. Risk factors for clustering and LP were investigated with partial proportional odds and logistic regression. Covariates included transmission mode, HIV-1 subtype, age, origin and cluster activity.

**Results:**

We included 1,040 individuals in the analysis, 59.6% identified with subtype B and 48.4% in a cluster. Risk factors for clustering included Danish origin (odds ratio (OR): 2.95; 95% confidence interval (CI): 2.21–3.96), non-LP (OR: 1.44; 95% CI: 1.12–1.86), and men who have sex with men (MSM). Increasing age and non-B subtype infection decreased risk (OR: 0.69; 95% CI: 0.50–0.94). Risk for late presentation was lower for active clusters (OR: 0.60; 95% CI: 0.44–0.82) and Danish origin (OR: 0.43; 95% CI: 0.27–0.67). Non-Danish MSM had a lower risk than non-Danish heterosexuals (OR: 0.34; 95% CI: 0.21–0.55).

**Conclusion:**

HIV-1 transmission in Denmark is driven by early diagnosed, young, subtype B infected MSM. These may benefit most from PrEP. Non-Danish heterosexual HIV-1 patients could benefit from improved communication to achieve earlier diagnosis and treatment.

## Introduction

The use of highly active antiretroviral therapy (HAART) has substantially increased the survival of people infected with HIV-1 [1,2] and has also been used to prevent transmission between partners [3,4] and from mother to child [5]. Pre-exposure prophylaxis (PrEP) has been shown to be both effective in reducing the number of new HIV-1 infections among people at risk [6] as well as being cost-effective [7]. Despite the benefits of PrEP, the incidence rates of HIV-1 in the World Health Organization (WHO) European Region have remained unaltered at 8.3–8.4 new HIV cases diagnosed per 100,000 inhabitants per year between 2008 and 2017 [8], primarily driven by steady incidence rates in non-European Union/European Economic Area countries. In Denmark, the rate has decreased slightly from 5.2 to 4.3 per 100,000 inhabitants per year during the same time period. Recent phylogenetic investigations have provided new insights into the dynamics and drivers of local transmissions [9-12], including that the Danish national transmissions are still mainly caused by HIV-1 subtype B [11]. In order to reduce the number of new HIV-1 infections, more knowledge is needed about which risk groups are causing this persistent transmission.

Patients who present late with HIV-1, the so-called late presenters (LP) are defined as patients with CD4^+^ T-cell count below 350 cells/µL or the presence of an acquired immunodeficiency syndrome (AIDS)-defining illness upon HIV diagnosis [13]. Their prevalence has previously been reported to vary between 38.3% to 49.8% in different European countries [14] and is currently at 49% in the WHO European Region [8]. In Denmark, the prevalence of LP is ca 50.5% and is higher among heterosexual HIV-1 patients (67.3%) compared with homosexual HIV-1 patients (34.9%) [8]. Despite their high prevalence, LP in Denmark were not found to contribute substantially to the ongoing transmission of HIV-1 in Europe [15]. However, late presentation with HIV-1 is a missed opportunity for early therapy initiation which is associated with increased risk of non-infectious multi-morbidities [16] and mortality [17].

In order to devise effective public health strategies aimed at reducing the ongoing transmission of HIV-1 and the high LP prevalence, knowledge about risk profiles and characteristics of national HIV-1 transmitters and LP is crucial. Furthermore, identifying these risk factors will inform targeted promotion and application of PrEP, behavioural or other intervention strategies to those with a high likelihood of transmission and those who benefit from early therapy. Similarly, better identification of vulnerable groups at risk for late presentation with HIV-1 would allow for improved targeting of HIV-1 screening strategies.

The aim of this study was to identify HIV-1 transmission clusters in Denmark between 2009 and 2017 by phylogenetic analysis; and to investigate whether origin (here used as born in Denmark or not), age, transmission mode, presentation status, and/or HIV-1 subtype are risk factors associated with being in a national cluster and whether these risk factors have changed during the study period. Correlates of late presentation of HIV-1 with any of these risk factors were also investigated.

## Methods

### Study population and characteristics

Blood samples from 1,225 newly diagnosed HIV-1 patients between 2009 and 2017, along with clinical and epidemiological information, were sent to Statens Serum Institut in Copenhagen, Denmark from infectious disease and HIV treatment centres as part of the long-running HIV-1 surveillance SERO project [18].

The SERO project forms the Danish sentinel framework for surveillance of transmitted HIV-1 resistance and molecular epidemiology [19,20]. Participation is voluntary and can include an analysis of the first sample within a year from diagnosis from newly diagnosed HIV-1 patients with no prior history of antiviral therapy.

Genotypic characterisation of the pol gene (protease and reverse transcriptase) was performed, as described previously [21]. Inclusion criteria were HIV-1 positive patients with a serum sample obtained no later than 6 months after the first positive HIV test conducted in Denmark and no previous history of HAART. Presentation status was assigned to patients in accordance with the consensus definition [13]: patients with a CD4^+^ T-cell count below 350 cells/µL or with an AIDS-defining illness, regardless of CD4^+^ T-cell count, were classified as LP; all others were designated as non-late presenters (NLP). In a sub-analysis, and in addition to the above groups, we defined patients with a CD4^+^ T-cell count below 200 cells/µL as very late presenters (VLP). Exclusion criteria were patients with unknown origin, transmission mode or infection status. Furthermore, we focused on sexual transmission and thus excluded people who inject drugs (PWID), transmission through blood transfusion or other transmission of non-sexual nature. When several possible transmission modes were stated, we assigned them to one category in the following overriding order: men who have sex with men (MSM) over PWID over blood transfusion over heterosexual HIV-1 patients (HSX). Whenever bisexual contact was mentioned as transmission mode, we considered this to be MSM. There were 17 patients with more than one transmission route, all HSX, and of these nine were excluded from the study.

**Figure 1:**
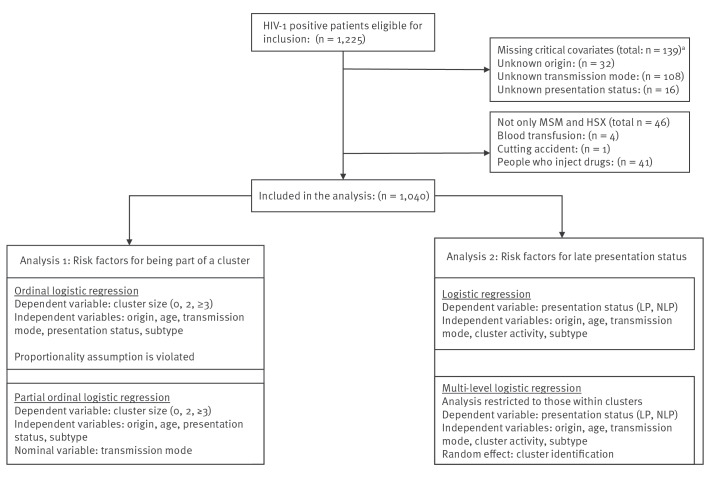
Flowchart of study population selection and statistical analyses, Denmark, 2009–2017

HIV-1: human immunodeficiency virus 1; HSX: heterosexual HIV-1 patients; LP: late presenters; MSM: men who have sex with men; NLP: non-late presenters.

^a^ Excluded HIV-1 patients can fall under multiple categories therefore the sum of the categories does not add up to the total displayed.

Patients registered as both male and female were assumed to be of the male sex for the purpose of the statistical analysis; no gender neutral patients were included in the study. Figure 1 presents the selection of the study population.

### Data analysis

#### Transmission cluster identification

The pol sequences from the commonly observed subtypes A (n = 100), B (n = 713), C (n = 83) and circulating recombinant forms (CRF)01 (n = 124) and CRF02 (n = 84) were aligned separately in Mafft version 6.0 [22] and phylogenetic analysis was performed using maximum likelihood with the general time reversible model with 100 bootstrap replicates in Mega 6.0 [22]. Less common subtypes and CRF (n = 121) were aligned together. Clusters were identified with Cluster Picker [23] using the initial and main support threshold of 0.9 and a genetic distance of 4.5. Transmission clusters were classified as active if they contained a patient sampled between 2015 and 2017; all other clusters were considered inactive in terms of HIV-1 transmission. For analysis of cluster size, we differentiated between no cluster, two patients in a cluster and clusters with three or more. Other groupings were tested but did not add relevant information (these are not shown here and included no cluster and clusters of two, three to four, five to six and seven or more; two, three to four and five or more; two, three and four or more; as well as two, three, four to five, six to 10 and 11 or more).

#### Genetic distance

To test dependence of our results on the genetic distance used in the clustering algorithm in Cluster Picker analysis, we also tested genetic distances of 1, 1.5, 3.0 and 4.5 using only subtype B sequences. Lowering the genetic distance used for clustering groups, means that sequences with a smaller genetic distance will still be grouped and these might indicate transmissions closer in time. Using various genetic distances may thus give an idea about the clusters with higher transmission rates.

#### Statistical analysis

The analyses consisted of two parts. In the first analysis we focussed on risk factors for being part of a cluster. In the second analysis we focused on risk factors for LP status.

#### Risk factors for being part of a cluster

To identify risk factors for being part of a cluster, the associations between cluster size, as identified by the phylogenetic analysis, and potential risk factors were investigated using ordinal logistic regression. More specifically, we fitted partial proportional odds models with a logit link and transmission mode as nominal effect. For this we used the cumulative link model function of the R ordinal package version 2015.6–28 (R Foundation, Vienna, Austria). Covariates included transmission mode (MSM or HSX), HIV-1 subtype (subtype B vs non-B subtype), presentation status (NLP and LP), age at the time of sample collection, period of diagnosis (2009–14 vs 2015–17), and origin (Danish origin or non-Danish origin).

#### Risk factors for late presentation status

Risk factors for LP status were analysed using logistic regression. Transmission mode, HIV-1 subtype, age at the time of sample collection, origin and cluster activity were included as covariates. The latter variable distinguished between no cluster, clusters with recent transmission that contained a sample from the period 2015 to 2017 and older clusters which contained no sample from 2015 to 2017. Since patients who are part of a cluster are more likely to have shared characteristics, it is possible that the risk estimates are biased. To test for this bias, we also performed a subset analysis using only patients within clusters. In this subset we tested for the same associations using a multi-level logistic regression model with cluster id as the random effect and compared the results to a naïve logistic regression model without random effects. Interclass correlation coefficients were calculated.

For both analyses we also investigated the potential role of interactions between covariates. Statistical significance of covariates and interactions were identified by the 95% confidence intervals (CI) for the odds ratio (OR) along with p values. Best fitting models were based on AIC-values and associated chi-squared model comparison tests in the case of nested models as well as the statistical significance of included coefficients. All analyses were performed in R version 3.3.3 (R Foundation, Vienna, Austria).

### Ethical statement

According to the Danish Act on Research Ethics Review of Health Research Projects, this study does not require approval by an ethics committee as it does not cause increased health risk or discomfort to patients. This was confirmed by the Committee on Health Research Ethics for the Region of Southern Denmark in a specific waiver of approval (VF20020258). Data were collected, stored and analysed as approved by the Danish data protection agency (J.nr. 2015–57–0102).

## Results

### Study population and descriptive statistics

The study population consisted of 1,225 patients, of which 1,040 (85%) were eligible for analysis given completeness of the associated epidemiological information (Table 1 and Figure 1). This corresponded to 46.6% of all cases of HIV-1 reported in Denmark between 2009 and 2017.

**Table 1:** Characteristics of HIV-1 patients by origin and presentation status, Denmark, 2009–2017 (n = 1,040)

Characteristics	Total (n = 1,040)		Danish origin (n = 675)				Non-Danish origin (n = 365)			
			NLP (n = 383)		LP (n = 292)		NLP (n = 158)		LP (n = 207)	
	n	%^a^	n	%^a^	n	%^a^	n	%^a^	n	%^a^
**Sex**										
Male	858	82.5	354	92.4	274	93.8	118	74.7	112	54.1
Female	182	17.5	29	7.6	18	6.2	40	25.3	95	45.9
**Average age (years)**	39.3	11.7	38.1	10.7	45.4	12.6	34.6	9.7	36.6	10.1
**HIV subtype**										
A	73	7.0	11	2.9	18	6.2	22	13.9	22	10.6
B	620	59.6	279	72.8	204	69.9	75	47.5	62	30.0
C	68	6.5	13	3.4	11	3.8	14	8.9	30	14.5
CRF01	112	10.8	40	10.4	26	8.9	17	10.8	29	14.0
CRF02	67	6.4	18	4.7	10	3.4	11	7.0	28	13.5
Other	100	9.6	22	5.7	23	7.9	19	12.0	36	17.4
**Transmission mode**										
MSM	651	62.6	303	79.1	192	65.8	91	57.6	65	31.4
HSX	389	37.4	80	20.9	100	34.2	67	42.4	142	68.6
**Cluster size**										
Not clustered	537	51.6	139	36.3	148	50.7	94	59.5	156	75.4
2	157	15.1	57	14.9	40	13.7	32	20.3	28	13.5
3	44	4.2	19	5.0	10	3.4	6	3.8	9	4.3
4–5	72	6.9	34	8.9	28	9.6	6	3.8	4	1.9
6–10	84	8.1	41	10.7	31	10.6	8	5.1	4	1.9
≥ 11	146	14.0	93	24.3	35	12.0	12	7.6	6	2.9
**Cluster activity**										
No cluster	537	51.6	139	36.3	148	50.7	94	59.5	156	75.4
Not active	192	18.5	82	21.4	59	20.2	25	15.8	26	12.6
Active cluster	311	29.9	162	42.3	85	29.1	39	24.7	25	12.1
**Period of diagnosis** ^b^										
2009–2011	405	38.9	139	34.3	135	46.2	52	12.8	79	38.2
2012–2014	327	31.4	126	38.5	87	29.8	61	18.7	53	25.6
2015–2017	308	29.6	118	38.3	70	24.0	45	14.6	75	36.2

CRF: circulating recombinant form; HIV-1: human immunodeficiency virus 1; HSX: heterosexual HIV-1 patients; LP: late presenters; MSM: men who have sex with men; NLP: non-late presenters.

^a^ Percentages may not all add up to 100% because of rounding.

^b^ Proportion represents the distribution of groups within the study period. One sample from 2008 was included.

Men constituted 82.5% of the study population and MSM was the most common (62.6%) mode of HIV-1 transmission. The MSM group was composed of 76.0% (495/651) men with a Danish origin, whereas 46.3% (180/389) of the HSX group had Danish origin. Late presentation with HIV-1 (LP or VLP) was observed among 48.0% (499/1,040) of the study population and 48.4% (503/1,040) of the study population was identified in clusters, with 29.0% (146/503) of these in clusters composed of 11 or more patients. Active clusters constituted 61.8% (311/503) of all identified clusters. Subtype B was the most common among those with Danish origin, while those of non-Danish origin showed greater variability in subtypes. In addition, those of Danish origin were more often part of clusters than patients of non-Danish origin.

### Risk factors associated with patients in national transmission clusters

**Figure 2:**
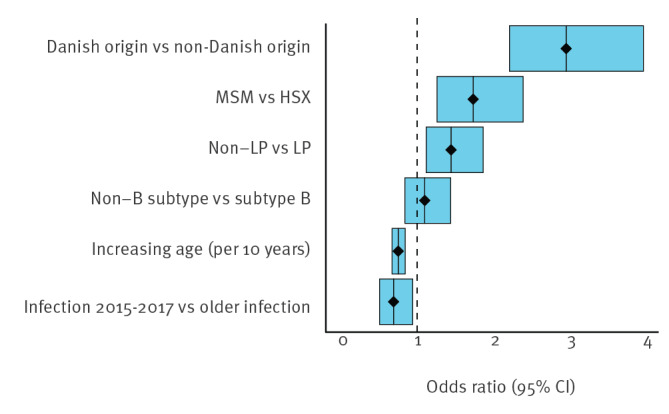
Risk factors for HIV-1 patients for being part of a cluster vs not being part of a cluster, Denmark, 2009–2017^a^ (n = 1,040)

CI: confidence interval; HIV-1: human immunodeficiency virus 1; HSX: heterosexual HIV-1 patients; LP: late presenters; MSM: men who have sex with men; NLP: non-late presenters.

^a^ Results are based on partial ordinal logistic regression.

The odds of being in any size category of cluster were higher for patients of Danish origin compared with those of non-Danish origin (OR: 2.95; 95% CI: 2.21–3.96; p < 0.00), Figure 2. Non-late presenters had higher odds of being part of a cluster than LP (OR: 1.44; 95% CI: 1.12–1.86; p < 0.00) and those with non-B subtype infections had lower odds of being part of a cluster (OR: 0.69; 95% CI: 0.50–0.94; p = 0.02). Overall, MSM had higher odds of being part of a cluster compared with HSX and the odds were higher for cluster size of two to three or more (OR: 2.47; 95% CI: 1.70–3.59) than for a cluster size of two or more to no cluster (OR: 1.46; 95% CI: 1.04–2.03). The odds of being part of any cluster decreased with age (OR: 0.97; 95% CI: 0.96–0.98; p < 0.00 for 1-year age bands). Patients sampled between 2015 and 2017 did not have higher odds of being part of a cluster (OR: 1.10; 95% CI: 0.84–1.44; p = 0.49). There were no statistically significant interaction terms. Model predictions of the probability of being part of a cluster are given in Supplementary Figure S1.

### Risk factors associated with late presentation status

Similar risk factors were investigated for late presentation status. Patients in active clusters with recent transmission events showed a considerably lower risk of being LP than those not part of a cluster (OR: 0.60; 95% CI: 0.44–0.82; p < 0.00), while patients in a non-active cluster did not show a higher risk compared with patients that were not part of a cluster (OR: 0.79; 95% CI: 0.56–1.14; p = 0.21).

**Figure 3:**
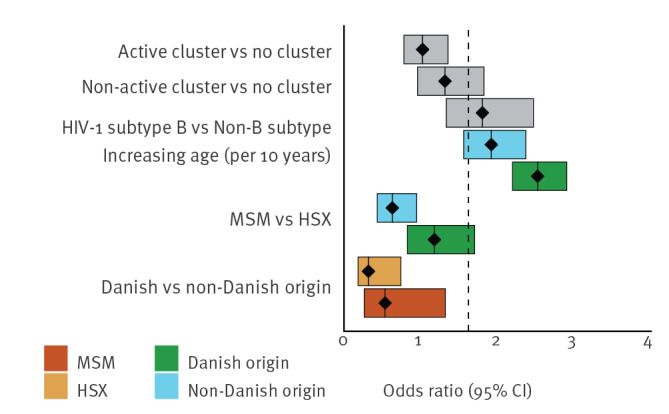
Risk factors of HIV-1 patients for being a late presenter vs non-late presenter, Denmark 2009–2017^a^ (n = 1,040)

CI: confidence intervals, HIV-1: human immunodeficiency virus 1; HSX: heterosexual HIV-1 patients; MSM: men who have sex with men.

^a^ Results are based on logistic regression.

In general, the odds of late presentation were lower for those of Danish origin (OR: 0.43; 95% CI: 0.27–0.67). In addition, there was a significant interaction between origin and transmission mode where generally Danish MSM had a significantly lower risk of being LP than non-Danish MSM (OR: 0.28; 95% CI: 0.10–0.80). However, the odds of being LP was higher for MSM vs HSX of Danish origin (OR: 0.71; 95% CI: 0.47–1.05) compared with the odds of MSM vs HSX of non-Danish origin (OR: 0.34; 95% CI: 0.21–0.55). In addition, the odds of being LP increased more with age for those of Danish origin compared with those of non-Danish origin (OR: 1.03; 95% CI: 1.00–1.06; p = 0.04). For example, a 10-year increase in age corresponds to an increase in the odds of being LP of 1.60 (95% CI: 1.38–1.85) for Danish origin and 1.20 (95% CI: 0.96–1.50) for non-Danish origin. Subtype B infection was not a significant risk factor for late presentation status (OR: 1.12; 95% CI: 0.81–1.56; p = 0.50). See also Figure 3.

To check for influences of clustering, we focused on cases within clusters (n = 503). In this sub-group analysis, only age and transmission mode were statistically significant with ORs of 1.03 (95% CI: 1.01–1.05; p < 0.00) and 0.54 (95% CI: 0.36–0.82; p < 0.00) respectively. Correcting for cluster id in a multi-level analysis did not meaningfully change our results, with corrected ORs of 1.04 (95% CI: 1.02–1.06; p < 0.00) and 0.55 (95% CI: 0.35–0.86; p < 0.00) for age and transmission mode, respectively. The intra-class correlation coefficient was 0.07, indicating a small correlation within clusters and thus lending support for our overall analyses above.

In the main analyses, VLP were included in the LP group and subsequent analyses did not show a statistical distinction between LP and VLP for the association with cluster size (p = 0.62). However, HSX were more at risk of being VLP (OR: 2.87; 95% CI: 1.72–4.89; p < 0.00) than MSM.

### Genetic distance

Table 2 shows descriptive statistics of patients clustered according to genetic distance cut-off points of 4.5, 3.0, 1.5, and 1.0. Only cluster size and cluster activity showed statistically significant differences between genetic distances. An association with cluster size is to be expected as reducing the genetic distance naturally decreases the number of larger clusters that may be more spread out in time and thus have larger genetic distances; indeed, there were proportionally more clusters of size ≥ 11 and fewer of smaller sizes at genetic distance 4.5 compared with smaller genetic distances. Contrary to our expectations, there were proportionally more active clusters at genetic distance 4.5 than at genetic distances 3.0 and 1.0. When comparing the model results for HIV subtype B between various genetic distances, the main difference is seen at genetic distance 1.5, where more recent infections are associated with being part of a cluster (see Supplementary Table S1 and S2).

**Table 2:** Characteristics of HIV-1 patients infected with subtype B and who are part of a cluster by genetic distance, Denmark, 2009–2017 (n = 352)

Characteristics	Genetic distance							
	4.5 (n = 352)		3.0 (n = 225)		1.5 (n = 106)		1.0 (n = 56)	
	n	%	n	%	n	%	n	%
**Sex**								
Male	337	95.7	215	95.6	102	96.2	56	100
Female	15	4.3	10	4.4	4	3.8	0	0
**Average age (years)**	38.1	11.0	37.6	10.9	36.5	10.4	35.2	10.4
**Transmission mode**								
MSM	312	88.6	201	89.3	98	92.5	54	96.4
HSX	40	11.4	24	10.7	8	7.5	2	3.6
**Danish origin**								
Yes	295	83.8	193	85.8	96	90.6	47	83.9
No	57	16.2	32	14.2	10	9.4	9	16.1
**Presentation status**								
NLP	219	62.2	141	62.7	71	67.0	41	73.2
LP	133	37.8	84	37.3	35	33.0	15	26.8
**Cluster size**								
2	85	24.1^a^	83	36.9^a,b^	55	51.9^a,b^	36	64.3^a,b^
3	27	7.7^a^	41	18.2^a,b^	23	21.7^a,b^	14	25.0^a,b^
4–5	57	16.2^a^	46	20.4^a,b^	4	3.8^a,b^	0	0.0^a,b^
6–10	50	14.2^a^	43	19.1^a,b^	24	22.6^a,b^	6	10.7^a,b^
≥ 11	133	37.8^a^	12	5.3^a,b^	0	0.0^a,b^	0	0.0^a,b^
**Cluster activity**								
Not active	130	36.9^c^	127	56.4^c^	56	52.8	34	60.7^c^
Active	222	63.1^c^	98	43.6^c^	50	47.2	22	39.3^c^
**Period of diagnosis**								
2009–2011	121	34.4	71	31.6	29	27.4	19	33.9
2012–2014	127	36.1	84	37.3	35	33.0	19	33.9
2015–2017	104	29.5	70	31.1	42	39.6	18	32.1

HIV-1: human immunodeficiency virus 1; HSX: heterosexual HIV-1 patients; MSM: men who have sex with men.

^a,b,c^ Superscripts indicate that these groups showed a statistical significant difference between each other at the Bonferroni corrected alpha of 0.0012.

## Discussion

In this study we found that 48.4% of patients diagnosed with HIV-1 in Denmark were part of a cluster and of those, 29.0% were part of a cluster containing more than 11 individuals. Furthermore, 61.8% of the clusters were identified as being active. Our analysis identified that patients of Danish origin, MSM, NLP, and those infected with subtype B, had higher odds for being part of a cluster among HIV infected patients and that the odds decreased with age. This suggests that HIV-1 transmission in Denmark is to a large extent driven by young MSM who are diagnosed early with HIV-1 subtype B.

Age has also been observed as an important factor in ongoing HIV-1 transmission in other studies [12,24]. Our finding that a substantial part of HIV-1 transmission occurs within networks of MSM infected with subtype B has also been reported in studies from other European countries, including Portugal [9,12]. In Denmark, the HIV epidemic has gradually become driven by subtype B infections.

Non-Danish origin seemed to be more related to smaller clusters or no-clusters, indicating that some of these patients may have been infected abroad and contribute to Danish clusters only to a limited degree. Interestingly, diagnosis between 2015–17 vs earlier period was not a significant risk-factor in our study, suggesting that there is no propagation in the number or size of clusters. In general, the risk profile for being part of a cluster is not surprising but does highlight a clear profile for those most at risk. Although comparable risk factors for national transmission have been identified in other European countries, recent studies from Spain [25] and Italy [26] show that newly introduced non-B subtypes can be established and transmitted relatively quickly. This highlights the importance of ongoing national surveillance in order to identify changes in the epidemic.

In total, 48% of the patients in the study were characterised as LP. Late presentation was significantly associated with not being part of an active cluster, age, and origin (overall, patients of Danish origin had lower odds of being LP). Our analysis identified HSX of non-Danish origin that were not part of an active cluster and individuals of Danish origin for whom the risk also increases with age as high-risk groups for LP status. These risk profiles are not unexpected, since transmission in Denmark is to a large extent driven by clusters of young MSM of Danish origin. These patients generally have full access to the Danish healthcare system and are thus more likely to be tested regularly and in case of infection be detected early. In contrast, those of non-Danish origin are more often infected abroad or immigrate to Denmark while already infected. This group is more commonly mixed, with a large proportion having been infected following HSX contact. Our finding that those in clusters are less likely to be LP indicates that those who are part of clusters are more likely to be tested for HIV-1 than those not in clusters, who may have a lower risk awareness of HIV. Interestingly, the odds of being a LP increased with age. This may indicate that young patients are testing more regularly compared with older patients or that those of higher age simply have had more time to become a LP.

Choice of genetic distance does not seem to have impacted our analysis. While several differences were found between genetic distance of 4.5 and 3.0, smaller genetic distance may indicate a closer time of diagnosis and thus can help identify highly active transmission clusters or superspreader events. However, the link between genetic distance and time is complex and we were not able to find clear differences between various genetic distances. Indeed, the choice of genetic distance heavily influences the size of clusters identified. For example, a genetic distance of 1.5 was unable to identify any of the large clusters of 11 or more patients in our data. When looking into the larger clusters more closely (see Supplementary Figure S2), these often seemed to consist of sub-clusters that could be identified when using smaller genetic distances.

These differences seem to imply that larger clusters with a genetic distance of 3.0 or more might depict a continuously growing transmission chain, whereas smaller clusters with a small genetic distance show the subset of transmission events more closely spaced in time. Contrary to our expectations, a smaller genetic distance was also associated with relatively fewer active clusters. It is possible that the sub-clusters are more often inactive, which implies that a few patients connect smaller clusters with one another over longer periods of time. Alternatively, it could indicate more recent changes in the dynamics of HIV-1 transmission in Denmark towards longer time intervals between infection events. However, in the current study, it was not possible to identify the reasons for this unexpected finding and future studies are needed to address this further.

While our findings in this molecular-epidemiological study are consistent with previous research, there are several limitations. First, this study was based on 46.6% of all newly diagnosed HIV-1 patients in the specified study period. This may have led to identifying fewer individuals belonging to clusters, especially in the case of smaller clusters. Since HIV is a notifiable disease in Denmark, all patients included in the SERO project are also reported to the national surveillance system. However, there are higher proportions of males (82.2% vs 75.3%), MSM (54.4% vs 46.9%) and persons of Danish origin (60.9% vs 47.6%) included in the SERO project compared with what was reported to the national HIV surveillance system during the 2009–17 period. In addition, the epidemiological information was collected through a self-reported questionnaire, which could have impacted the accuracy of some answers. While the questionnaire included questions about risk behaviour, it did not include questions about the number of sex partners. Since the answers to these questions were often missing, they were not included in the analysis and therefore residual confounding may be present in our results. Furthermore, information on HIV positive test results is requested as part of the routine SERO survey, albeit responses on test outcomes are rarely provided. Active sentinel surveillance systems like this are commonly used for surveillance purposes in cases where it is not possible to obtain a more comprehensive surveillance system, for instance if participation is voluntary.

Another possible limitation is that we used the consensus definition for late presentation [13], which has been shown to overrepresent the proportion of LP [27]. Since HIV negative or positive test dates were only available for a small proportion of patients in this study, other means of establishing time since infection were not possible. In addition, since the consensus definition is commonly used, its use in this study will also make the results more comparable to other studies.

Despite these limitations, our analysis highlights the added value of HIV-1 surveillance systems, such as the SERO project, to public health monitoring, especially when they include the sampling of biological material. Such systems allow for more detailed analysis, which can improve the active control of HIV-1 spread, through identification of potential clusters, monitoring of the development of these clusters as well as specifically targeting the risk groups involved with intervention and communication strategies.

### Conclusion

Our study identified several target groups for PrEP and communication/advice on PrEP or behavioural intervention strategies. Specifically, those in active clusters with young Danish MSM are key in the ongoing transmission of HIV in Denmark and targeting this group with PrEP/behavioural interventions may achieve the most benefit in reducing the ongoing national transmission. Also, we identified that HSX of non-Danish origin that are not part of a transmission cluster are at a high risk to present late for HIV-diagnosis and may therefore benefit from improved communication and information about the benefits of an early diagnosis and treatment initiation.

## Supplementary Data

447000 Supplement

## References

[1] Antiretroviral Therapy Cohort Collaboration . Life expectancy of individuals on combination antiretroviral therapy in high-income countries: a collaborative analysis of 14 cohort studies. Lancet. 2008;372(9635):293-9. 10.1016/S0140-6736(08)61113-7 18657708 PMC3130543

[2] Teeraananchai S Kerr SJ Amin J Ruxrungtham K Law MG . Life expectancy of HIV-positive people after starting combination antiretroviral therapy: a meta-analysis. HIV Med. 2017;18(4):256-66. 10.1111/hiv.12421 27578404

[3] Cohen MS Chen YQ McCauley M Gamble T Hosseinipour MC Kumarasamy N Prevention of HIV-1 infection with early antiretroviral therapy. N Engl J Med. 2011;365(6):493-505. 10.1056/NEJMoa1105243 21767103 PMC3200068

[4] Cohen MS Chen YQ McCauley M Gamble T Hosseinipour MC Kumarasamy N Antiretroviral therapy for the prevention of HIV-1 transmission. N Engl J Med. 2016;375(9):830-9. 10.1056/NEJMoa1600693 27424812 PMC5049503

[5] Connor EM Sperling RS Gelber R Kiselev P Scott G O’Sullivan MJ Reduction of maternal-infant transmission of human immunodeficiency virus type 1 with zidovudine treatment. Pediatric AIDS clinical trials group protocol 076 study group. N Engl J Med. 1994;331(18):1173-80. 10.1056/NEJM199411033311801 7935654

[6] Chou R Evans C Hoverman A Sun C Dana T Bougatsos C Preexposure prophylaxis for the prevention of HIV infection: evidence report and systematic review for the US preventive services task force. JAMA. 2019;321(22):2214-30. 10.1001/jama.2019.2591 31184746

[7] Cambiano V Miners A Dunn D McCormack S Ong KJ Gill ON Cost-effectiveness of pre-exposure prophylaxis for HIV prevention in men who have sex with men in the UK: a modelling study and health economic evaluation. Lancet Infect Dis. 2018;18(1):85-94. 10.1016/S1473-3099(17)30540-6 29054789 PMC5988036

[8] European Centre for Disease Prevention and Control (ECDC) and World Health Organization. (WHO) Regional Office for Europe. HIV/AIDS surveillance in Europe 2018–2017 data. Copenhagen: WHO Regional Office for Europe; 2018. Available from: https://www.ecdc.europa.eu/en/publications-data/hivaids-surveillance-europe-2018-2017-data

[9] Pineda-Peña AC Pingarilho M Li G Vrancken B Libin P Gomes P Drivers of HIV-1 transmission: The Portuguese case. PLoS One. 2019;14(9):e0218226. 10.1371/journal.pone.0218226 31568476 PMC6768452

[10] Fearnhill E Gourlay A Malyuta R Simmons R Ferns RB Grant P A phylogenetic analysis of human immunodeficiency virus type 1 sequences in Kiev: findings among key populations. Clin Infect Dis. 2017;65(7):1127-35. 10.1093/cid/cix499 28575385 PMC5850412

[11] Petersen A Cowan SA Nielsen J Fischer TK Fonager J . Characterisation of HIV-1 transmission clusters and drug-resistant mutations in Denmark, 2004 to 2016. Euro Surveill. 2018;23(44):1700633. 10.2807/1560-7917.ES.2018.23.44.1700633 30401010 PMC6337072

[12] Paraskevis D Beloukas A Stasinos K Pantazis N de Mendoza C Bannert N HIV-1 molecular transmission clusters in nine European countries and Canada: association with demographic and clinical factors. BMC Med. 2019;17(1):4. 10.1186/s12916-018-1241-1 30616632 PMC6323837

[13] Antinori A Coenen T Costagiola D Dedes N Ellefson M Gatell J Late presentation of HIV infection: a consensus definition. HIV Med. 2011;12(1):61-4. 10.1111/j.1468-1293.2010.00857.x 20561080

[14] Mocroft A Lundgren J Antinori A Monforte Ad Brännström J Bonnet F et al. Late presentation for HIV care across Europe: update from the collaboration of observational HIV epidemiological research Europe (COHERE) study, 2010 to 2013. Euro Surveill. 2015;20(47):30070. 26624933 10.2807/1560-7917.ES.2015.20.47.30070

[15] Audelin AM Cowan SA Obel N Nielsen C Jørgensen LB Gerstoft J . Phylogenetics of the Danish HIV epidemic: the role of very late presenters in sustaining the epidemic. J Acquir Immune Defic Syndr. 2013;62(1):102-8. 10.1097/QAI.0b013e318276becc 23075917

[16] Guaraldi G Zona S Menozzi M Brothers TD Carli F Stentarelli C Late presentation increases risk and costs of non-infectious comorbidities in people with HIV: an Italian cost impact study. AIDS Res Ther. 2017;14(1):8. 10.1186/s12981-016-0129-4 28209189 PMC5311843

[17] Pacheco PRG Zara ALSA Silva E Souza LC Turchi MD . Late onset of antiretroviral therapy in adults living with HIV in an urban area in Brazil: prevalence and risk factors. J Trop Med. 2019;2019:5165313. 10.1155/2019/5165313 31080478 PMC6475541

[18] Audelin AM Lohse N Obel N Gerstoft J Jørgensen LB . The incidence rate of HIV type-1 drug resistance in patients on antiretroviral therapy: a nationwide population-based Danish cohort study 1999-2005. Antivir Ther. 2009;14(7):995-1000. 10.3851/IMP1412 19918103

[19] van de Laar MJ Bosman A Pharris A Andersson E Assoumou L Ay E Piloting a surveillance system for HIV drug resistance in the European Union. Euro Surveill. 2019;24(19):1800390. 10.2807/1560-7917.ES.2019.24.19.1800390 31088600 PMC6518967

[20] Petersen A Cowan SA Nielsen J Fischer TK Fonager J . Characterisation of HIV-1 transmission clusters and drug-resistant mutations in Denmark, 2004 to 2016. Euro Surveill. 2018;23(44):1700633. 10.2807/1560-7917.ES.2018.23.44.1700633 30401010 PMC6337072

[21] Abdissa A Yilma D Fonager J Audelin AM Christensen LH Olsen MF Drug resistance in HIV patients with virological failure or slow virological response to antiretroviral therapy in Ethiopia. BMC Infect Dis. 2014;14(1):181. 10.1186/1471-2334-14-181 24708645 PMC4234735

[22] Tamura K Stecher G Peterson D Filipski A Kumar S . MEGA6: Molecular evolutionary genetics analysis version 6.0. Mol Biol Evol. 2013;30(12):2725-9. 10.1093/molbev/mst197 24132122 PMC3840312

[23] Ragonnet-Cronin M Hodcroft E Hué S Fearnhill E Delpech V Brown AJL Automated analysis of phylogenetic clusters. BMC Bioinformatics. 2013;14(1):317. 10.1186/1471-2105-14-317 24191891 PMC4228337

[24] Pineda-Peña A-C Theys K Stylianou DC Demetriades I Abecasis AB Kostrikis LG . HIV-1 infection in Cyprus, the Eastern Mediterranean European frontier: a densely sampled transmission dynamics analysis from 1986 to 2012. Sci Rep. 2018;8(1):1702. 10.1038/s41598-017-19080-5 29374182 PMC5786036

[25] Delgado E Benito S Montero V Cuevas MT Fernández-García A Sánchez-Martínez M Diverse large HIV-1 non-subtype B clusters are spreading among men who have sex with men in Spain. Front Microbiol. 2019;10:655. 10.3389/fmicb.2019.00655 31001231 PMC6457325

[26] Fabeni L Alteri C Berno G Scutari R Orchi N De Carli G Characterisation of HIV-1 molecular transmission clusters among newly diagnosed individuals infected with non-B subtypes in Italy. Sex Transm Infect. 2019;95(8):619-25. 10.1136/sextrans-2019-054017 31076456

[27] Sasse A Florence E Pharris A De Wit S Lacor P Van Beckhoven D Late presentation to HIV testing is overestimated when based on the consensus definition. HIV Med. 2016;17(3):231-4. 10.1111/hiv.12292 26222266 PMC5034831

