# Supplementary material for: Identification of risk factors associated with national transmission and late presentation of HIV-1, Denmark, 2009 to 2017
Source: Euro Surveill. 2021 Nov 25;26(47):2002008. doi: 10.2807/1560-7917.ES.2021.26.47.2002008 (PMC8619873; doi:10.2807/1560-7917.ES.2021.26.47.2002008)

This supplementary material is hosted by *Eurosurveillance* as supporting information alongside the article “Identification of risk factors associated with national transmission and late presentation of HIV-1, Denmark, 2009 to 2017”, on behalf of the authors, who remain responsible for the accuracy and appropriateness of the content. The same standards for ethics, copyright, attributions and permissions as for the article apply. Supplements are not edited by *Eurosurveillance* and the journal is not responsible for the maintenance of any links or email addresses provided therein.

Supplementary information to:

"Identification of risk factors associated with national transmission and late presentation of HIV-1, Denmark, 2009 to 2017"

Authors: Maarten van Wijhe[1,2], Thea Kølsen Fischer[3,4], Jannik Fonager[2]

1. Department of Science and Environment, Roskilde University, Roskilde, Denmark

2. Virus Research & Development Laboratory, Virus & Microbiological Special Diagnostics, Statens Serum Institut, Copenhagen, Denmark

3. Department of Research, University hospital of Nordsjælland, Hillerød, Denmark

4. Department of Public Health, University of Copenhagen, Copenhagen, Denmark

Corresponding author: Jannik Fonager (fon@ssi.dk)

Contents

1. Table S1: Model results for the association between cluster size and explanatory variables.
2. Table S2: Model results for the association between presentation status and explanatory variables.
3. Figure S1: Model estimates of the probability of HIV infected patients to be part of a cluster
4. Figure S2: Analysis of genetic distances varying between 1.0 and 4.5

Table S1: Model results for the association between cluster size and explanatory variables. For the comparison of genetic distances only patients infected with HIV subtype B are used.

	Odds ratio (95% confidence interval)				
	**Main results**	**Genetic distance (restricted to HIV subtype B)**			
		**4.5**	**3.0**	**1.5**	**1.0**[a]
Age	097 (0.96 – 0.98)	0.98 (0.96 – 0.99)	097 (0.96 – 0.99)	0.97 (0.95 – 0.99)	0.96 (0.93 – 0.99)
Danish					
No	Ref	Ref	Ref	Ref	Ref
Yes	2.95 (2.21 – 3.96)	2.78 (1.90 – 4.10)	2.71 (1.75 – 4.30)	4.07 (2.10 – 8.73)	1.81 (0.89 – 4.11)
Infection mode (nominal effect)					
HSX	Ref	Ref	Ref	Ref	-
MSM, cluster of 2+ vs no cluster	1.46 (1.04 – 2.03)	1.50 (0.94 – 2.40)	1.39 (0.83 – 2.33)	1.86 (0.85 – 4.08)	-
MSM cluster of 3+ vs cluster of 2	2.47 (1.70 – 3.59)	1.42 (0.87 – 2.32)	1.16 (0.64 – 2.10)	1.63 (0.56 – 4.75)	-
Presentation status					
LP	Ref	Ref	Ref	Ref	Ref
NLP	1.44 (1.12 – 1.86)	1.34 (0.98 – 1.85)	1.19 (0.84 – 1.68)	1.34 (0.85 – 2.15)	1.79 (0.97 – 3.45)
Year					
2009-2014	Ref	Ref	Ref	Ref	Ref
2015-2017	1.10 (0.84 – 1.44)	1.30 (0.92 – 1.83)	1.36 (0.94 – 1.95)	2.26 (1.43 – 3.55)	1.27 (0.68 – 2.28)
Subtype					
B	Ref	-	-	-	-
Non-B	0.69 (0.50 – 0.94)	-	-	-	-

[a]The model with infection mode as a nominal effect did not converge for a genetic distance of 1 due to low counts among HSX. Results show the model with infection mode excluded, care must be taken in interpreting this model.

Table S2: Model results for the association between presentation status and explanatory variables. For the comparison of genetic distances only patients infected with subtype B are used. Interaction terms were not considered in these models.

	Odds ratio (95% confidence interval)				
	**Main results**	**Genetic distance (restricted to HIV subtype B)**			
		**4.5**	**3.0**	**1.5**	**1.0**
Age	1.02 (0.99 – 1.04)	1.04 (1.02 -1.06)	1.05 (1.02 – 1.08)	1.03 (0.98 – 1.08)	1.01 (0.94 – 1.07)
Danish					
No	Ref	Ref	Ref	Ref	Ref
Yes	0.13 (0.04 – 0.41)	0.72 (0.39 – 1.32)	0.74 (0.33 – 1.70)	1.74 (0.38 – 12.48)	1.04 (0.19 – 8.01)
Infection mode					
HSX	Ref	Ref	Ref	Ref	Ref
MSM	0.34 (0.21 – 0.55)	0.75 (0.37 – 1.51)	0.92 (0.36 – 2.37)	0.73 (0.13 – 4.12)	0.60 (0.02 – 19.36)
Cluster activity					
Inactive	Ref	Ref	Ref	Ref	Ref
Active	0.76 (0.52 – 1.11)	0.74 (0.47 – 1.16)	0.71 (0.40 – 1.26)	0.45 (0.19 – 1.05)	0.31 (0.06 – 1.17)
No- cluster	1.25 (0.88 – 1.79)	-	-	-	-
Subtype					
B	Ref	-	-	-	-
Non-B	0.89 (0.64 – 1.24)	-	-	-	-
Interactions					
*Age*Danish*	1.03 (1.00 – 1.06)	-	-	-	-
*Infection mode*Danish*	2.07 (1.17 – 3.69)	-	-	-	-

Figure S1: Model estimates of the probability of HIV infected patients to be part of a cluster. Cluster was defined as: not in a cluster (red), in a cluster of 2 (blue), and in a cluster of 3 or more (green). Estimates are based on partial proportional odds ordinal logistic regression with logit link and transmission mode as nominal effect. Covariates included transmission mode (MSM: Men who have sex with Men, HSX: Heterosexual), HIV-1 subtype (Subtype B or non-B subtype), presentation status (LP: late presenter, NLP: non-late presenter), age at time of sample collection, ethnicity (Danish , non-Danish), and recent transmission (2009-2014, 2015-2017). Shown here are the estimates restricted to 2015-2017.

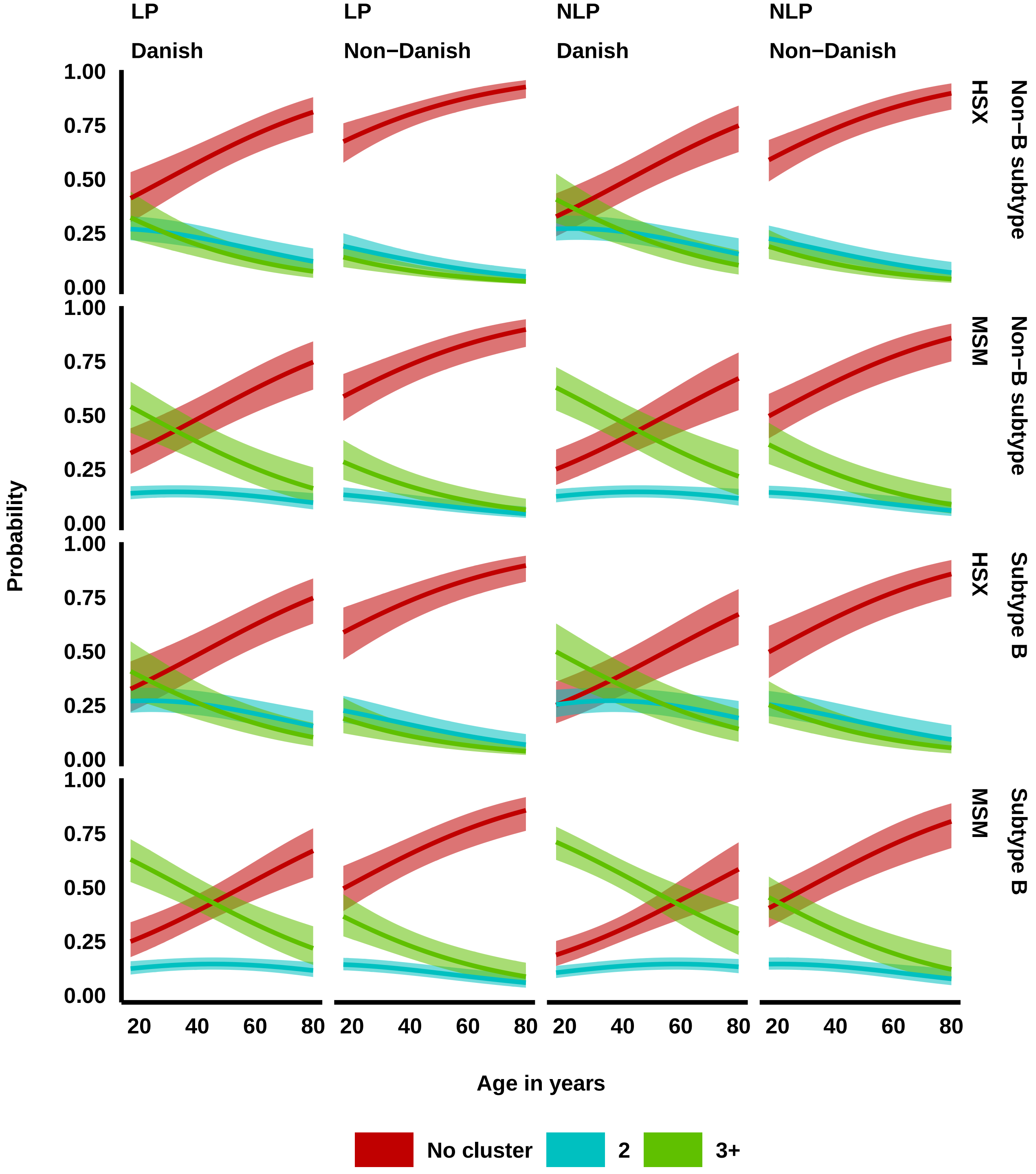

Figure S2: Analysis of genetic distances varying between 1.0 and 4.5. Only HIV-1 Subtype B are used in this analysis and for representation purposes only clusters of size 3+ are shown. Each individual cluster is connected by a line, panels indicate the clusters identified at varying genetic distances (also indicated by colour). Comparing the panels from bottom to top gives an idea how the identified clusters change when using increasingly lower thresholds for genetic distance. Clusters are ordered by first sampling date in the cluster.

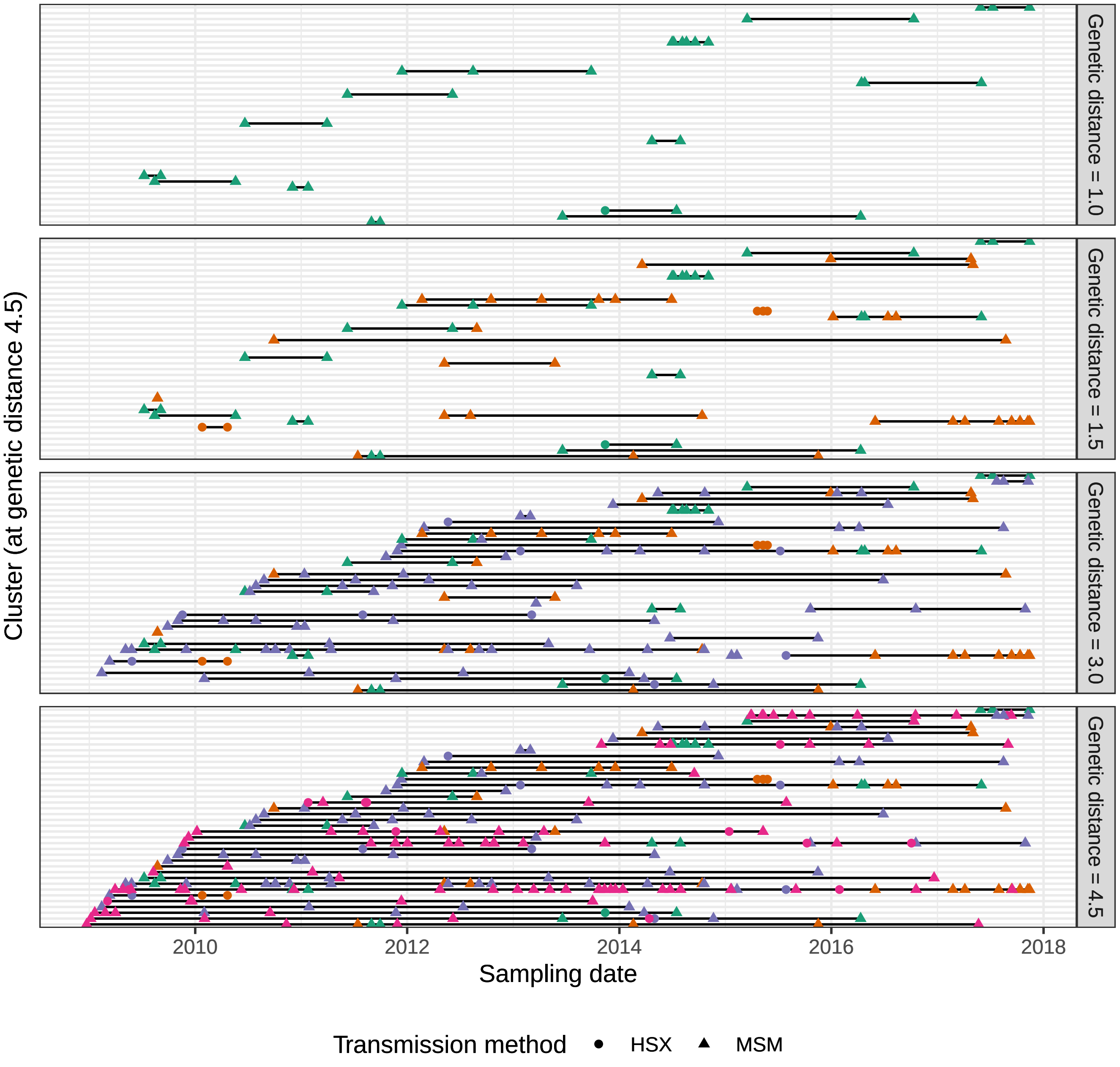

Supplement: Supplement [file 20-02008_FONAGER_Supplementary_Material.pdf]
